# Effect of Microsize and Nanosize TiO_2_ on Porous Mullite-Alumina Ceramic Prepared by Slip Casting

**DOI:** 10.3390/ma17246171

**Published:** 2024-12-17

**Authors:** Ludmila Mahnicka-Goremikina, Maris Rundans, Vadims Goremikins, Ruta Svinka, Visvaldis Svinka, Liga Orlova, Inna Juhnevica

**Affiliations:** 1 Institute of Materials and Surface Engineering, Faculty of Natural Science and Technology, Riga Technical University, Paula Valdena st. 3/7, LV-1048 Riga, Latvia; 2 Institute of Civil Engineering, Faculty of Civil and Mechanical Engineering, Riga Technical University, Kipsalas st. 6A, LV-1048 Riga, Latvia

**Keywords:** slip casting, porosity, thermal expansion, mullite, titanium dioxide

## Abstract

Sintered porous mullite-alumina ceramics are obtained from the concentrated suspension of powdered raw materials such as kaolin, gamma and alpha Al_2_O_3_, and amorphous SiO_2_, mainly by a solid-state reaction with the presence of a liquid phase. The modification of mullite ceramic is achieved by the use of micro- and nanosize TiO_2_ powders. The phase compositions were measured using an X-ray powder diffraction (XRD) Rigaku Ultima+ (Tokyo, Japan) and microstructures of the sintered specimens were analysed using scanning electron microscopy (SEM) Hitachi TM3000-TableTop (Tokyo, Japan). The shrinkage, bulk density, apparent porosity, and water uptake of the specimens was determined after firing using Archimedes’ principle. The apparent porosity of the modified mullite ceramic is 52–69 ± 1%, water uptake is 33–40 ± 1%, pore size distributions are 0.05–0.8 μm, 0.8–10 μm and 10–1000 μm, and bulk density are variated from 1.15 ± 0.05 to 1.4 ± 0.05 g/cm^3^. The microsize TiO_2_ and nanosize TiO_2_ speed up the mullitisation process and allow the decrease in the quantity used as raw material amorphous SiO_2_, which was the purpose of the study. The use of nanosize TiO_2_ additive increases the porosity of such a ceramic, decreasing the bulk density and linear thermal expansion.

## 1. Introduction

The production of mullite and mullite-corundum ceramics is based on the use of aluminium oxide and silicon oxide raw materials and their sintering. Crystalline mullite is difficult to obtain from powdered raw materials due to slow ion diffusion. Ghate, Dokko, and Sacks in 1975, 1977, and 1982, respectively, determined that the activation energy E_a_ of mullitisation is 800–1400 kJ/mol and it is at its peak at 1000 °C. The activation energy of mullitisation depends on the activation energy of mullite nucleation and crystal growth, as well as on the relatively high diffusion activation energy of Si^4+^ ions in the Al_2_O_3_-SiO_2_ system (Ea = 702 kJ/mol) [1,2].

On completion of the mullitisation reaction, sintering and complete densification of mullite occurs at temperatures >1700 °C due to relatively slow mutual diffusion of Si^4+^ and Al^3+^ ions [1,3,4]. The mullitisation kinetics strongly depend on the methods of synthesis of mullite, raw materials, type of sample preparation and firing temperature. Various strategies and methods are being investigated to reduce the formation temperature of the mullite phase. For example, mullite can form at 1400–1500 °C from Al_2_O_3_ particles covered with an amorphous silicon oxide layer. The growth and sintering of mullite grains can be accelerated by reducing the particle size of the raw materials or by reducing the diffusion distance between aluminium and silicon ions by increasing the densification level of the raw materials. By using Al_2_O_3_ nanoparticles, the mullitisation temperature can be lowered to 1300 °C [3,4,5].

α-Al_2_O_3_ and γ-Al_2_O_3_ are usually used as alumina raw materials, and quartz, cristobalite and amorphous SiO_2_ are used as silica raw materials. The formation and sintering of mullite from these raw materials are a multi-step process that, depending on the type of raw materials, consists of nucleation of mullite, the rapid mullitisation stage and slow mullitisation stage, and finally, the stage with a high rate of mullite formation [2,4]. Kaolinite clay, namely kaolin, is an important raw material for the production of mullite and plays an important role in reducing the cost of obtaining mullite ceramics.

Kaolin is a relatively prevalent soft, often chalk-like sedimentary rock with an earthy smell. Kaolin is low-plasticity clay, and the melting point of pure kaolin is around 1770 °C, while impurities lower its melting point [2,5]. The main mineral of these clays is kaolinite. The theoretical formula of kaolinite is Al_2_O_3_·2SiO_2_·2H_2_O. Kaolinite is formed in the kaolinisation process from feldspar (potassium feldspar), granite or aluminium silicates over a long period of time. It is known from the literature that the percentage of the main oxides of kaolin from different deposits can vary up to 10 wt%. Thus, for example, the content of SiO_2_ and Al_2_O_3_ is 53.7 wt% and 41.8 wt% in French kaolin, 53.1 wt% and 44.4 wt% in Algerian kaolin, 62.1 w t% and 36.8 wt% in Malayan kaolin, and 52.8 wt% and 44.7 wt% in American kaolin, respectively [1,6,7]. Kaolin also contains other oxides and minerals, the amount of which is variable, for example, 0.05 wt% of Fe_2_O_3_ and CaO, 0.04 wt% of MgO, Na_2_O and TiO_2_, and 0.03 wt% of K_2_O. Usually, kaolin additionally contains quartz and mica, and less often, feldspar, illite, montmorillonite, hematite, bauxite, zircon, rutile, kyanite, sillimanite and others [1,5,7].

When kaolinite clay is fired at temperatures from 1000 to 1300 °C, the liquid phase is not formed; instead, a compound with a molar ratio of aluminium and silicon oxides of 3:2 is obtained, which corresponds to the mullite [4,8].

Mullite ceramics can be modified with alkali metal oxides, alkaline earth metal oxides and transition metal oxides. These modifications have influenced the intensity of the mullitisation process and phases of mullite ceramics, ceramic morphology, microstructures, and other properties [3,9,10,11,12,13,14].

Significant replacement of Al^3+^ ions in the crystal structure of mullite is possible. Ti^4+^, V^3+^, Cr^3+^, Fe^3+^ and Ga^3+^ are the main substituting ions originating from transition metal oxide (TiO_2_, V_2_O_3_, Cr_2_O_3_, Fe_2_O_3_ and Ga_2_O_3_) additives, which, as a result of substitution, affect the parameters of the mullite lattice cell. The degree of ion replacement or solubility depends on the radii of transition cations and the degree of oxidation. Transition metal cations V^3+^, Cr^3+^, Fe^3+^, Ti^4+^, Ti^3+^ and others with ionic radii in the range of 0.54–0.62 Å replace Al^3+^ (0.53 Å) in [AlO_6_] octahedral sites. Substitution occurs up to a certain concentration [11,15,16,17].

Porous ceramic is the ceramic materials group with porosity over 30% that was created intentionally. The process of manufacturing porous ceramic includes replica techniques, sacrificial template, freeze-drying, foam-gelcasting and direct foaming. Each of these methods have their own advantages and disadvantages [18,19,20,21,22]. In the case of the replica method, the cellular structure involves impregnation with a ceramic suspension, removal of excess slurry, and pyrolysis of the combustible substrate. The result is macroporous ceramics with a morphology similar to the original porous material. In the case of the sacrificial template process, a sacrificial phase could be homogeneously dispersed throughout a continuous ceramic matrix. The difficulties of the first method exist because suspension impregnation limits the use of small cells, and the second method requires a uniform distribution of combustible particles. Also, both methods are characterized by a CO_2_ release during firing due to the decomposition of organic compounds [23,24]. Freeze-casting is a more environmentally friendly method for porous ceramics manufacturing, but requires equipment operations at temperatures lower than −18 °C, as well as the solvent freezing process control, such as cooling rate, sublimation of solvent crystals under reduced pressure, and a temperature of about −60 °C. Adjustments to the solid loadings of slurries influence the porous ceramics morphology [23,24,25,26,27,28,29]. Such a method like foam-gelcasting allows us to obtain porous ceramics with high apparent porosity, but requires the use of a gelling system, for example, using poly-γ-glutamic acid (γ-PGA) [30] or Isobam-104 (IB) [31], a foaming agent such as sodium dodecyl sulfate [30] or triethanolamine lauryl sulfate (TLS) [31] and foam stabilisation with sodium carboxymethyl cellulose (CMC) [31,32]. In this method the suspensions of ceramic raw materials can be aerated with a double shear mixer. The gelling agent addition is needed for foam consolidation after foaming by vigorously stirring with a mixer. The porous ceramic structure after foam-gelcasting preparation is characterized by shell foams with a network of spherical pores uniformly distributed in a matrix [32]. Obtaining porous ceramics via a simple preparation and environmentally friendly method occupies an important position. Such a method can be in the form of direct foaming and the use of a foaming agent, which is a commonly used method for the fabrication of porous ceramics. Direct foaming of the ceramic raw materials’ slurry occurs when the gaseous phase is released due to a chemical reaction of the components. In an example, H_2_O_2_, O_2_, air, CO_2_, NH_3_, H_2_ etc. can be used as blowing agents, and mechanical agitation can be used to produce bubbles in the ceramic slurry. In direct foaming, the thermodynamic stability and kinetics of bubble formation determine the ceramic porosity, pore size distribution and pore geometry [33,34,35].

The production of porous mullite and mullite-alumina ceramics remains topical and important nowadays due to their chemical resistance, thermal stability, mechanical strength and long durability [36]. The variation in pore size distribution, interconnectivity, permeability and microstructure of the porous mullite and mullite-alumina ceramic allow for different applications of their use [37,38]. Different porous mullite composite ceramic membranes and filters are used to interact uniquely with oil droplets in oil refining [39] and oil–water filtration [40,41], industrial wastewater treatment [40,42,43], arsenic removal from borehole water [44], mullite ceramic composite membranes for highly efficient CO_2_/N_2_ separation and stability at high temperatures [45], mullite fibre-based porous ceramic for catalytic carriers and ozonation process [46], and for heat insulation and sound absorption, for example, self-reinforcing porous mullite ceramics [47] or mullite fibrous porous ceramic [48].

In the case of this study, H_2_ was used as blowing agents that formed due to the chemical reaction of aluminium with water, as an environmentally friendly method [49]. Micro- and nano TiO_2_ were used for the investigation of the mullitisation of porous alumina-mullite ceramic with a high content of kaolin.

The mullite-alumina ceramics prepared through slip casting methods (concentrated suspension casting) are suitable for large-scale industrial application, because it is a low-cost method and does not contain complex technological processes or installations; however, it is important to take into account the rheological properties of the raw materials slurry. A national-level patent has also been granted for the use of the concentrated suspension casting technique to obtain highly porous ceramics [50].

## 2. Materials and Methods

### 2.1. Raw Materials and Samples’ Preparation

The main raw materials of prepared mullite ceramics were kaolin, α- and γ- Al_2_O_3_, and amorphous SiO_2_ in powder form. Kaolin (d_50_ = 1.5 μm) with 56.5 wt% of SiO_2_ and 31.0 wt% of Al_2_O_3_ was purchased from MEKA, Amberger Kaolinwerke, Germany, α-Al_2_O_3_ (d_50_ = 2 μm, Nabalox NO 725) and γ-Al_2_O_3_ (d_50_ = 80.0 μm, Nabalox NO 201) were purchased from Nabaltec AG, Schwandorf, Germany, and amorphous SiO_2_ (d_50_ = 3.0–5.0 μm) was purchased from GetNanoMaterials, Saint-Cannat, France. Micro- and nanosize TiO_2_ (GetNanoMaterials) with an average particle size of 5.0 μm and 20.0 nm, respectively, were used for mullite ceramic modification. Aluminium paste “Aquapor-9008” with a solid content of 70 ± 2% was purchased from Schlenk Metallic Pigments GmbH, Roth, Germany and used as a pore-forming agent for direct foaming.

The amount of kaolin was 30 pbw (parts by weight) and was served as a mullite formation agent as well as a binder agent for raw material suspension stabilisation. The ratio of α-Al_2_O_3_ to γ-Al_2_O_3_ was 1:3. The amount of SiO_2_ is half that of the SiO_2_ quantity in the stoichiometric 3Al_2_O_3_·2SiO_2_ mullite composition in order to reduce the use of this expensive raw material. Thus, the ratio of Al_2_O_3_ to SiO_2_ was 2.57:0.5. Microsize TiO_2_ was used in quantities of 1, 2.5 and 5 pbw, but nanosize TiO_2_ additive was set at 1 pbw. In all samples, 0.2 pbw of aluminium paste was used. Aluminium paste was used as a pore-forming agent due to the H_2_ evolution as the result of the reaction between the Al paste and H_2_O by the formula 2Al + 6H_2_O → 2Al(OH)_3_ + 3H_2_. Aluminium hydroxide decomposes during the sintering of A_2_O_3_ and H_2_O, and then the A_2_O_3_ reacts with SiO_2_. Table 1 summarises the raw material compositions of the samples.

Slip casting of concentrated slurry raw materials was the method used for porous ceramic forming. First, a slurry of starting materials was prepared, and then a slurry of aluminium paste was added. The water content of the prepared slurry was 40–45 pbw. The combined suspension was then mixed with a mechanical mixer for about 10 min. The presence of kaolin and uniform mixing by a laboratory mixer at a rotation speed of about 1000–1500 rpm allowed us to obtain a uniform distribution of all components in the final mix. The same viscosities of the suspensions were observed for all compositions during preparation. Thus, kaolin is both a component of mullite ceramics and a binder, which provides the necessary viscosity and uniform distribution of modifying oxide additives. Therefore, the mullite ceramic formation and modification was observed by the one-stage sintering process that is technically convenient and economically profitable. After slip-casting in the mould, the kaolin in viscous suspension prevents the lamination and sedimentation of modifying additives, thus, a uniform modified mullite ceramic structure is obtained after sintering.

**Figure 1:**
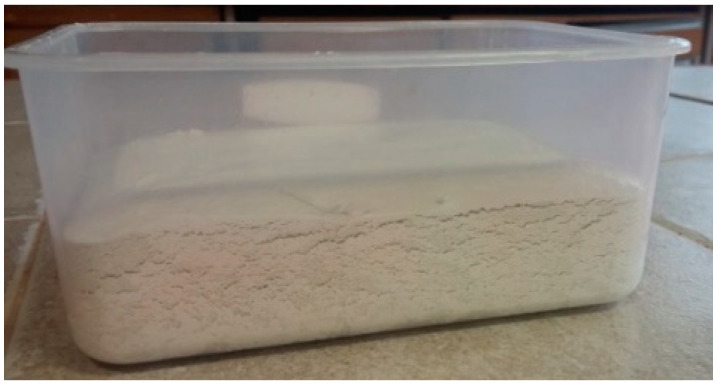
Solidified samples.

The formation of pores was not noticeable immediately after the pouring of the slurry. The pores became barely visible to the human eye after 30 min, but after an hour their size increased, and after two hours the pores became visible to the naked eye. Figure 1 shows the solidified samples.

As a result, the volume of the mould content increased 1.5 times. Samples were solidified and dried at room temperature for 24 h, after which samples were dried at 50 °C for about 48 h.

The dried samples were sintered at 1400 °C and 1500 °C with a 250 °C/h (4.2 °C/min) heating rate, and the holding time at maximum temperature was 1 h. The fired samples’ cooling process took place at the same rate as the heating process.

### 2.2. Characterisation

Appropriate equipment was used to analyse the properties of sintered samples. X-ray powder diffraction (XRD) was carried out with a Rigaku Ultima+ (Tokyo, Japan) diffractometer using Cu Ka radiation operating at 30 kV and 20 mA. XRD patterns were scanned in the 5–60 2θ° measurement angle range with a 0.02° step, and a 2°/min goniometer scanning rate was used to determine the crystalline phases. An automated Rietveld refinement method was used for quantitative phase analysis by using data obtained from X-ray diffraction analysis [51].

The shrinkage (*S*) after ceramics’ firing was calculated as a percentage using the mathematical Equation (1):

*S* = ((*L* − *L*_0_)/*L*_0_) × 100 (1)

where *L*_0_ is the original length and *L* is the length after firing.

The bulk density (*p*), apparent porosity (*P*) and water uptake (*Wup*) measurements were based on the Archimedes’ principle according to the European standard EN 623-2 [52], and water was used as a liquid medium. The dry weight of the samples (*w_d_*), the weights of the samples immersed in water (*w_i_*) and the weights of the materials suspended in water, (*w_w_*) were used for these calculations [53,54].

The bulk density was calculated from the following Equation (2):

*p* = *w_d_*/(*w_w_* − *w_i_*) × *p*_H_2_O_. (2)

The apparent porosity was calculated from the following Equation (3):

*P* = ((*w_w_* − *w_d_*)/(*w_w_* − *w_i_*)) × 100. (3)

The water uptake was calculated from the following Equation (4):

*Wup* = ((*w_w_* − *w_d_*)/*w_d_*) × 100. (4)

The mercury porosimeter (Quantachrome, Pore Master 33, Boynton Beach, FL, USA) was used to determine the pore size distribution of the porous mullite ceramic. The measured pore size range was from 0.1 μm to 1000 μm. The Hitachi TM3000-TableTop scanning electron microscope from Japan was used to investigate the structures of the sintered samples. The high temperature horizontal dilatometer L76/1600 D (Linseis, Selb, Germany) was used to determine the linear thermal expansion coefficient (LTEC) of the samples from 200 °C to 1300 °C.

## 3. Results and Discussion

### 3.1. X-Ray Diffraction and Scanning Electron Microscopy Results

**Figure 2:**
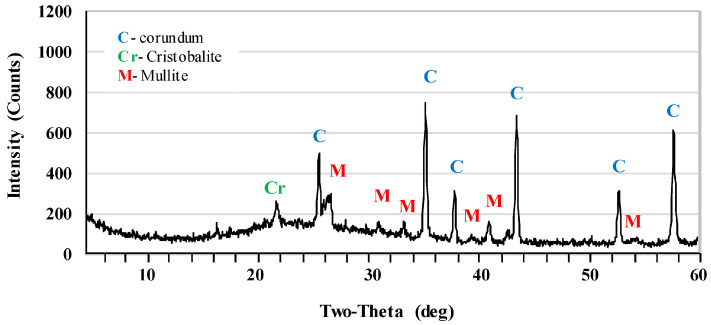
XRD patterns of the AK1.4 samples sintered at the 1400 °C.

**Figure 3:**
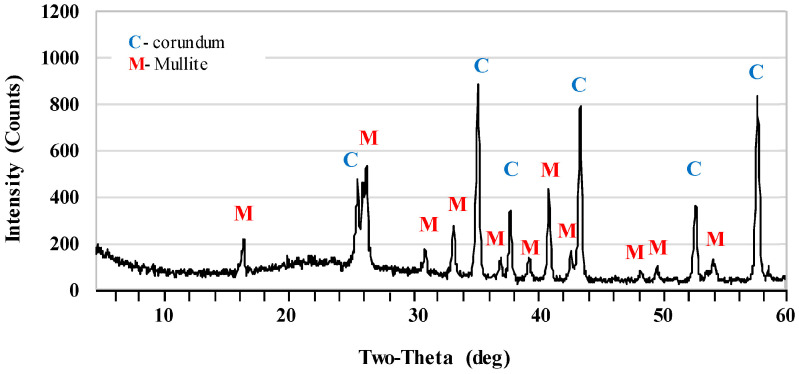
XRD patterns of the AK1.5 samples sintered at the 1500 °C.

**Figure 4:**
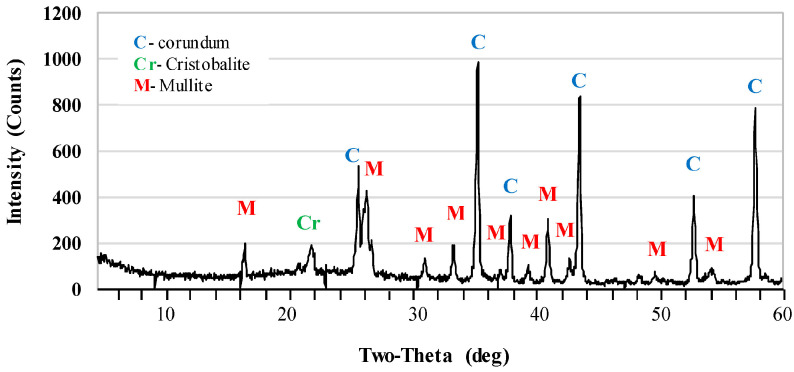
XRD patterns of the AK2 samples sintered at the 1500 °C.

**Figure 5:**
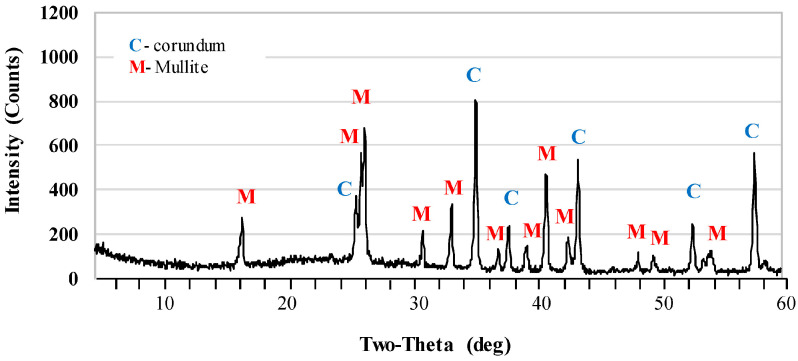
XRD patterns of the AK3 samples sintered at the 1500 °C.

**Figure 6:**
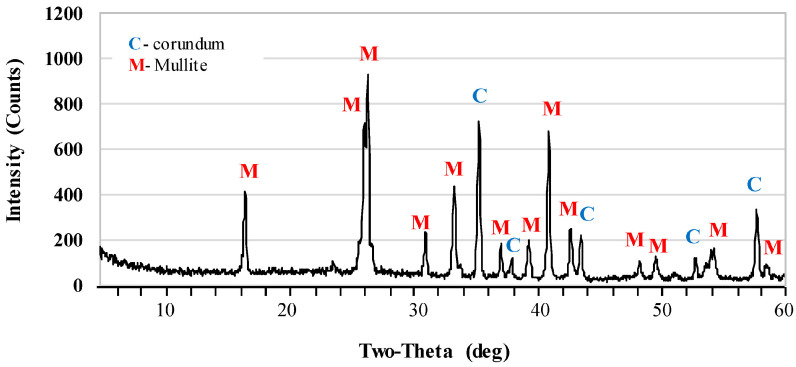
XRD patterns of the AK4 samples sintered at the 1500 °C.

**Figure 7:**
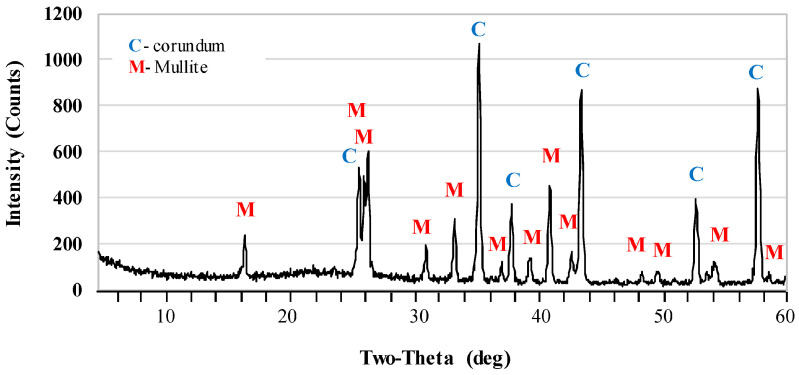
XRD patterns of the AK5 samples sintered at the 1500 °C.

Figure 2 and Figure 3 show the X-ray diffraction patterns of undoped samples sintered at temperatures of 1400 °C and 1500 °C. Corundum (alumina, α-Al_2_O_3_) was the main phase of samples that were sintered at the 1400 °C. Mullite and cristobalite were only weakly detected in these samples. In turn, the peak intensity of the mullite phase increased significantly after sintering at 1500 °C. The use of 1 pbw (AK2) of microsize TiO_2_ and sintering at 1500 °C did not affect the mullite phase intensity (Figure 4). AK2 samples consisted of 35% mullite, 62% corundum and 3% cristobalite (Table 2). Figure 5 and Figure 6 show that doping with 2.5 pbw (AK3) and 5 pbw (AK4) of microsize TiO_2_ and sintering at 1500 °C, significantly and proportionally increases the intensity of the mullite phase peaks. Quantitative phase compositions (Table 2) show that in case of the AK3 and AK4 samples, the mullite phase was dominant over the corundum phase; respectively, mullite takes up 60% and 85% accordingly; the remaining amount is corundum, as is evident in the decreased intensity of the corresponding corundum peaks. The use of 1 pbw of nanosize TiO_2_ and sintering at 1500 °C (AK5 sample) had a higher influence on the mullite formation than the use of 1 pbw of the microsize TiO_2_ (Figure 7). AK5 samples consist of 44% mullite and 56% corundum crystalline phase. Thus, TiO_2_ speeds up the mullitisation process, if dopant particles are extremely or relatively fine in large quantities, as is the case of 5 pbw of microsize TiO_2_. A separate TiO_2_ crystalline phase did not show on any of the X-ray diffraction patterns due to TiO_2_ being in the amorphous phase. In the case of the addition of the TiO_2_ in Al_2_O_3_–SiO_2_ system, a solid solution is generated due to the close radius of Ti^4+^ and Al^3+^. Thus, the Al^3+^ is replaced by Ti^4+^ and produces cationic vacancies. As a result, lattice distortion occurs, which are helpful for ion diffusion, accelerating mullitisation, and decreasing the sintering temperature. Therefore, TiO_2_ acts as a sintering additive and nucleating agent. The small amount of nanosize TiO_2_ increases the mullite nuclei, and development and growth of the crystals [55,56].

**Figure 8:**
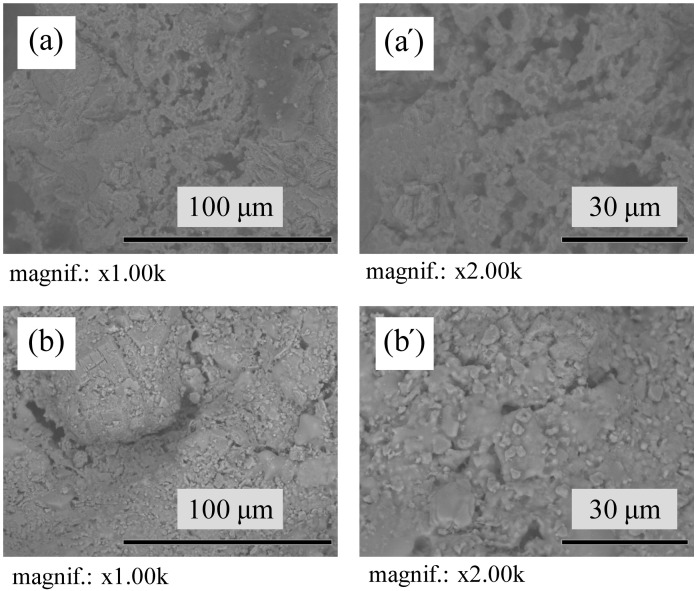
TableTop SEM micrographs of the microstructure of sintered undoped samples: (**a**,**a’**) AK1.4 sample; (**b**,**b’**) AK1.5 sample.

**Figure 9:**
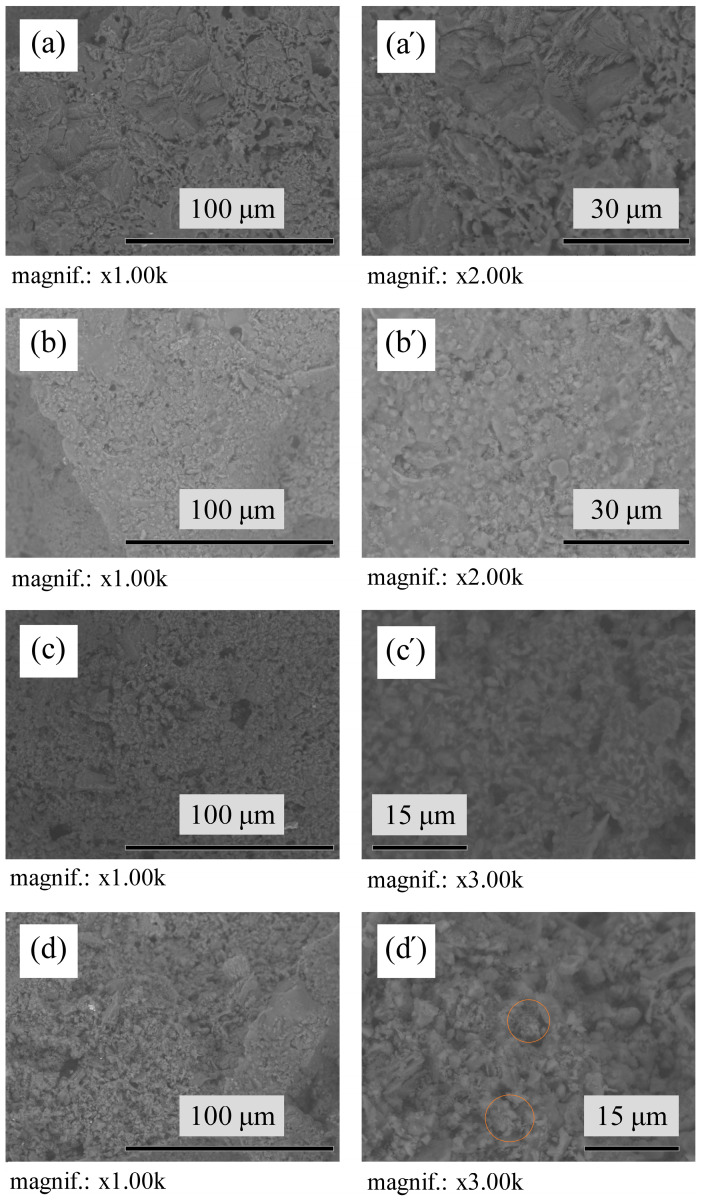
TableTop SEM micrographs of the microstructure of sintered modified samples: (**a**,**a’**) AK2 sample; (**b**,**b’**) AK3 sample; (**c**,**c’**) AK4 sample; (**d**,**d’**) AK5 sample.

The SEM micrographs of the microstructure of undoped samples sintered at 1400 °C and 1500 °C, shows the presence of ≈ 80 μm agglomerates of corundum due to the use of larger particles of γ-Al_2_O_3_, that convert to the α-Al_2_O_3_ during the firing step as a result of polymorphism [57] (Figure 8). The microstructure of all sintered samples that can be observed in Figure 8 and Figure 9 are mainly granular. Elongated needle-like mullite crystals evenly distributed in structure are observed in AK4 samples with 5 pbw of TiO_2_ (Figure 9c’). In the case of the AK5 samples, the mullite crystals are very small, but they can still be observed in the structure, marked with an orange circle (Figure 9d’).

### 3.2. Shrinkage and Bulk Density of the Sintered Samples

**Figure 10:**
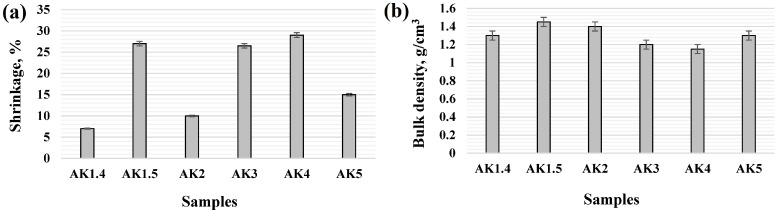
Characterisations of the samples: (**a**) shrinkage and (**b**) bulk density.

Figure 10 shows the shrinkage and bulk density of sintered samples. Increasing the sintering temperature of undoped samples from 1400 °C to 1500 °C increased the shrinkage from 7 ± 1% to 27 ± 1%. Samples with 1 pbw (AK2) of microsize TiO_2_ have the shrinkage of about 10 ± 1%, but samples with 2.5 pbw and 5 pbw of microsize TiO_2_ have about three times this value due to the intensive formation of the liquid phase in system TiO_2_-Al_2_O_3_-SiO_2_ which better consolidates the overall matrix. As is evident from the X-ray diffraction analysis, there are no traces of the TiO_2_ crystalline phase as one would expect (e.g., rutile, anatase or brookite), nor are there any variations of TiO_2_-containing aluminosilicate phases, which leads to a situation wherein all of the doped Ti^4+^ is either incorporated into the mullite/corundum crystalline lattice as a substitute or contained within the amorphous phase. The drastic increase in shrinkage of the doped samples suggests the preference for the latter. The samples with 1 pbw (AK5) of nanosize TiO_2_ have a shrinkage of about 15 ± 1%. Although the effect of the addition of 1 pbw-TiO_2_ nano is smaller in comparison to either the 2.5 or 5 pbw (samples AK3 and AK4) microsized TiO_2_ additive, it still accelerates the mullite formation more so than the 1% microsized TiO_2_ sample (AK2). The bulk density of the samples sintered at 1500 °C and doped with microsize TiO_2_ decreases from 1.4 ± 0.05 g/cm^3^ to 1.15 ± 0.05 g/cm^3^ with the mullite phase increasing in the sample compositions. The bulk density of the samples (AK5) with 1 pbw of nanosize TiO_2_ is 1.3 ± 0.05 g/cm^3^. The shrinkage and bulk density parameter difference between samples AK2 and AK5 can therefore be attributed to the dopant particle size difference, with smaller nanosize dopants being more reactive.

From Table 3, it can be seen that the shrinkage of various porous mullite specimens reported in the literature is close in meaning to the value of the obtained products. Respectively, it is about 21–22%, but bulk density is higher by 2–2.5 times [58]. The specifically used mullite fibres can prevent the shrinkage of porous mullite ceramics [59], that is by 10 times in comparison with obtained ceramics with a similar bulk density (1.109–1.385 g/cm^3^), but at a certain point, the increase in mullite fibre content causes the bulk density to increase. Also, the maximum sintering temperature in this case is noticeably higher.

Yu Chen et al. obtained porous mullite membranes with a porosity of 74.51%, low bulk density 0.80 g/cm^3^, and 21% of shrinkage by using a photosensitive slurry based on calcined kaolin with the addition of ZrSiO_4_ and Digital Light Processing (DLP) technology. Such a porous ceramic manufacturing method is multi-staged and quite energy-intensive [60]. The use of the optimal amount of 10 wt% lignin fibres as a burning additive for porous mullite ceramic preparation from mullite micropowder slurries by the gelcasting prevents drying shrinkage. As a result, it also decreases the full shrinkage [61], but the bulk density (1.48 g/cm^3^) of such a specimen is higher than for samples prepared with TiO_2_ in our case.

### 3.3. Apparent Porosity and Pore Size Distributions of the Sintered Samples

**Figure 11:**
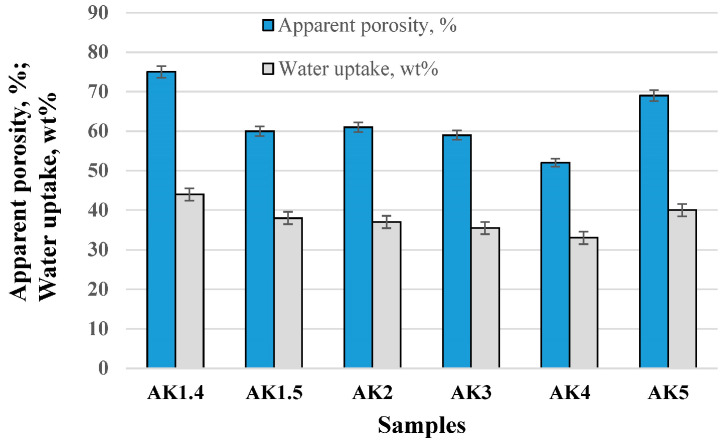
Apparent porosity and water uptake of the samples.

Figure 11 shows the apparent porosity and water uptake. Apparent porosity of the undoped samples decreased from 75 ± 1% to 60 ± 1%, with the sintering temperature increasing from 1400 °C to 1500 °C due to densification. The apparent porosity of doped samples with microsize TiO_2_ decreased from 60 ± 1% to 52 ± 1%, due to the increasing quantity of TiO_2_, and the sintering temperature increase. In the case of samples (AK5), nanosize TiO_2_ porosity is 69 ± 1%. The use of 1 pbw of 20 nm TiO_2_ increases the porosity of samples in comparison with samples with 1 pbw of microsize TiO_2_.

**Figure 12:**
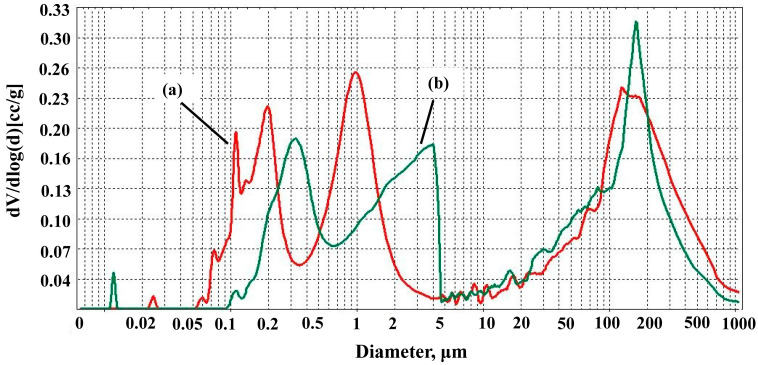
Pore size distributions of the samples: (a) AK1.4 sample, (b) AK1.5 sample.

**Figure 13:**
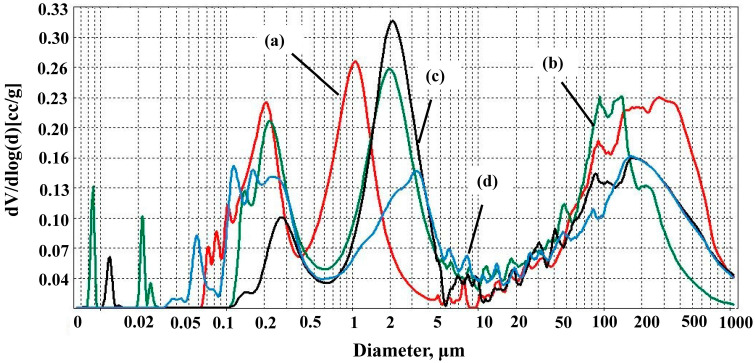
Pore size distributions of the samples: (a) AK2 sample, (b) AK3 sample, (c) AK4 sample, and (d) AK5 sample.

Figure 12 and Figure 13 show pore size distributions of samples. Undoped samples have three ranges of pore size distributions. Samples AK1.4 have pores from about 0.05–0.45 μm, 0.5–3 μm and pores of a larger size—from 20 μm to 1000 μm. In the case of A1.5 samples, the range of small pores has shifted towards large pores of about 0.1–0.7 μm and 0.7–5 μm due to sintering and disappearance of small pores after the firing of samples at 1500 °C. 

There are mainly three pore size distribution ranges for doped samples. The range of small pores has also shifted towards large pores with an increase in the used microsize TiO_2_ quantity. For AK2 samples, these ranges are about 0.05–0.5 μm, 0.5–5 μm, and large pores of about 10–1000 μm with intense pore sizes of about 120 μm. For AK3 and AK4 samples, the ranges are about 0.1–0.8 μm, and 0.8–10 μm and large pores are about 10–1000 μm in size. For AK5 samples, pores with the size of 0.05–0.8 μm, 0.8–10 μm and 10–1000 μm occupy approximately the same percentage of the volume in the sample.

**Figure 14:**
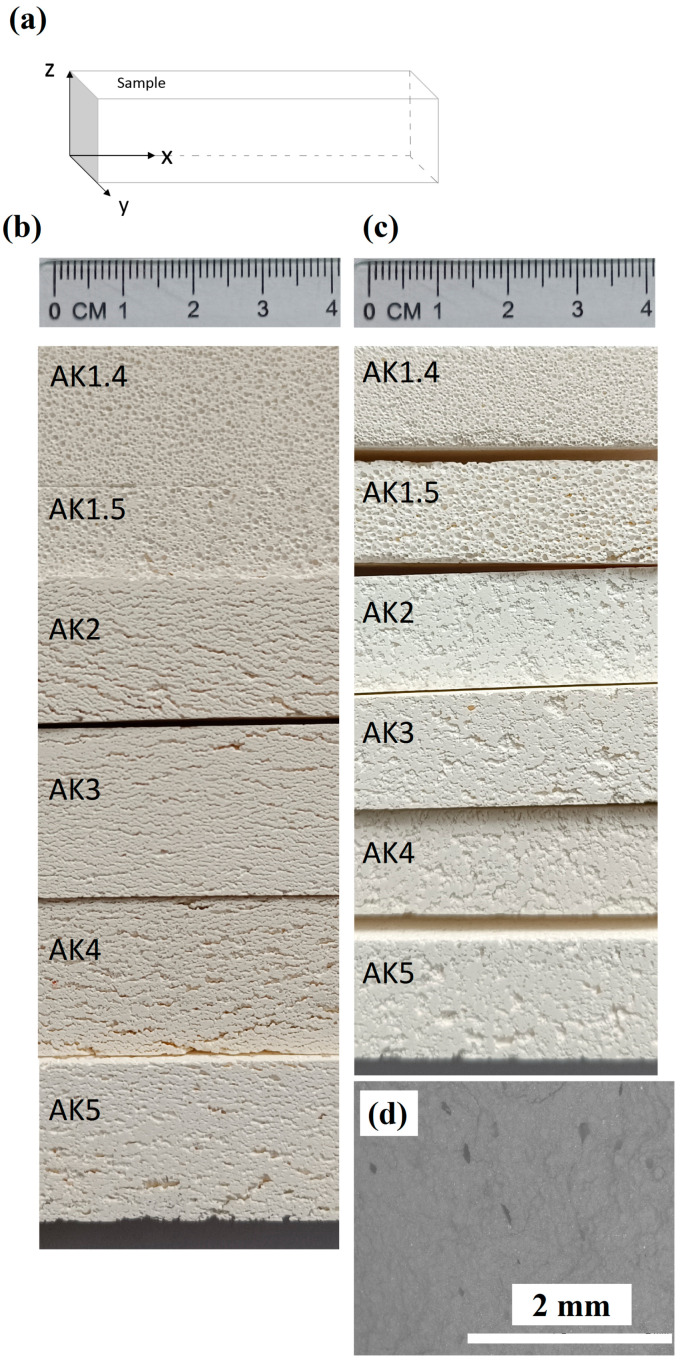
Illustration of sintered samples: (**a**) Sample planes; (**b**) photographs of samples, zx-plane; (**c**) photographs of samples, xy-plane; and (**d**) SEM micrograph of sample without bubbling, magnification ×50.

Figure 14a shows the samples planes and Figure 14b,c shows photographs of zx- and xy-planes of synthesized samples, where pores are visible to the naked eye. The SEM micrograph (Figure 14d) demonstrates the presence of some rare spherical pores in the synthesized undoped sample without the addition of the pore-forming agent (Al paste suspension). These pores could have formed during the mixing process of the suspension.

**Figure 15:**
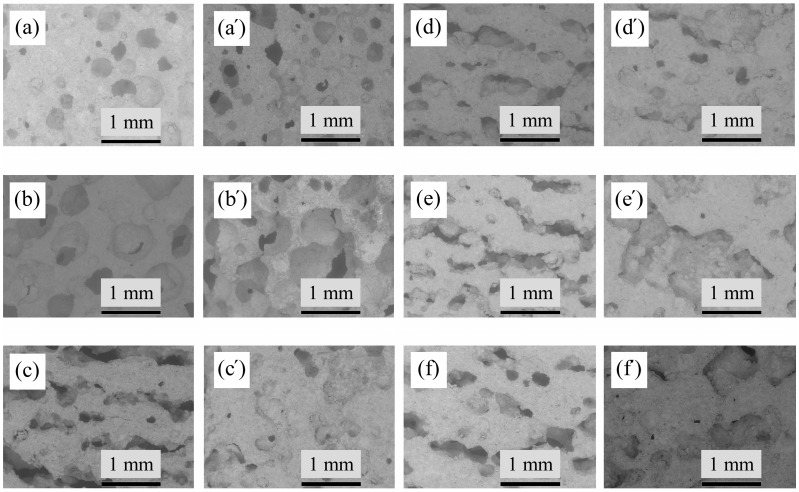
TableTop SEM micrographs illustrating the macrostructure of the samples, magnification ×50: (**a**–**f**) view of pores in the zx-plane; (**a’**–**f’**) view of pores in xy-plane; (**a**,**a’**) AK1.4 sample, (**b**,**b’**) AK 1.5 sample, (**c**,**c’**) AK2 sample, (**d**,**d’**) AK3 sample, (**e**,**e’**) AK4 sample, and (**f**,**f’**) AK5 sample.

Porous undoped samples AK1.4 and AK1.5 have spherical pores (Figure 15a–b’). Pores of TiO_2_-doped samples have elongated, oriented along the zx-plane, slightly branched pores (Figure 14b and Figure 15c–f’). The elongated pores are oriented parallel to the base of the sample and occur after pouring the suspension into the mould (Figure 1). The formation of elongated pores can be explained by the Ostwald ripening effect [62,63]. In the case of doped samples, after the slurry was poured into the moulds, it is possible to assume that pores formed with relatively small and wide distribution, but with time, larger bubbles formed and consumed smaller ones. Such a process occurs because larger bubbles are more energetically favoured than smaller bubbles. Ostwald ripening or coarsening is prevalent in the evolution of such a system as a gas–liquid foam. Pores combine in a mainly horizontal direction that is parallel to the base of the samples’ shape due to higher energy efficiency. Suspension mass pressure is directed downward towards the formed gas bubbles, due to the Earth’s gravity, therefore, it is easier for bubbles to coalesce in a horizontal direction than in a vertical direction. The decrease in the samples’ porosity with increasing amount of microsize TiO_2_ can be explained by the viscosity increase in the continuous slurry phase. As a result, the gas diffusion is lower, and the rate of gas transport is reduced, which causes the formation of the pores with a size range of 0.5–10 μm. In turn, the growth of bubbles slows with the increase in the suspension’s viscosity, due to the samples’ solidification that occurs when the bubbles form, which also influences the formation of elongated pores. In the case of the nanosize TiO_2_, it generates an adsorption layer with low gas permeability at the air–water interface, and can therefore maintain spherical air bubbles. Thus, the AK5 samples have pores of both elongated and rounded shapes, which is noticeable in Figure 15f,f’) [63,64].

Obtained modified porous mullite ceramics have a porosity of about 52–69%, which is relatively similar and, in some cases, even higher in comparison with other results from the latest reports, as can be seen in the Table 3. These ceramic samples are obtained by more simple, ecological methods that do not emit harmful gases. This is the case with burnable additives, which require specific substances and multi-stage formation, with firing at a temperature of 1500 °C, which in some cases is at least 100 °C lower, and a 2–4 times shorter holding time [58,59,60,61]. The pore form and size distribution of obtained modified samples significantly differ from the results of Table 3. The specific elongated, slightly branched pores form with three size diapasons (about 0.05–0.8 μm, 0.8–10 μm and 10–1000 μm) of obtained modified samples, giving them potential advantages in the use of ceramics as membranes for filtration or catalyst carriers, as well as thermal insulators.

### 3.4. Linear Thermal Expansion

**Figure 16:**
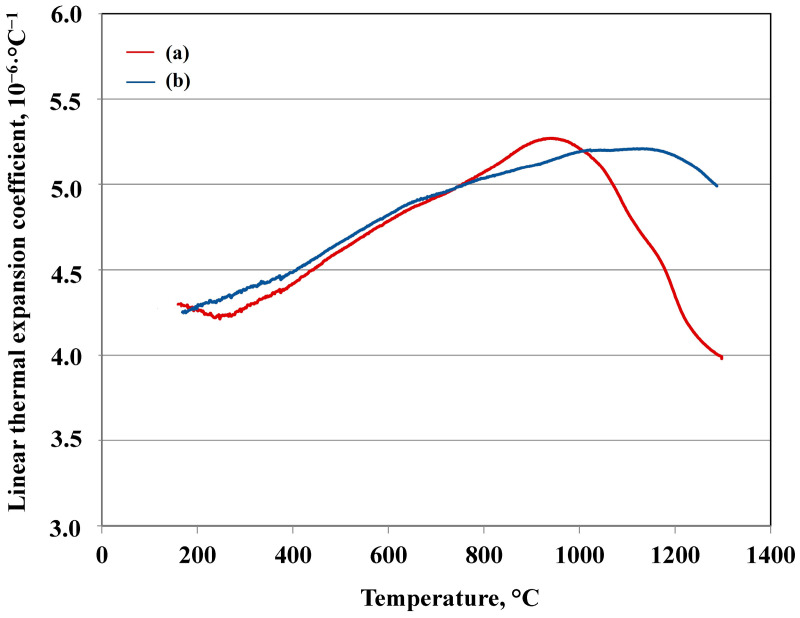
Temperature dependence of the linear thermal expansion coefficient for sintered samples: (a) of the undoped sample sintered at the 1500 °C; (b) of the sample with 1 pbw of nanosize TiO_2_.

Linear thermal expansion of the undoped sample sintered at 1500 °C and a sample with 1 pbw of nanosize TiO_2_ were analysed for comparison and are shown in Figure 16. The average linear thermal expansion coefficient of undoped samples was analysed from 200 °C to 800 °C and from 800 °C to 950 °C, and was 4.6·10^−6^·°C^−1^, and 5.2·10^−6^·°C^−1^, respectively. The average LTEC of doped 1 pbw nanosize TiO_2_ samples was also analysed from 200 °C to 800 °C, from 800 °C to 950 °C and from 950 °C to 1150 °C, and was 4.7·10^−6^·°C^−1^, 5.1·10^−6^·°C^−1^, and 5.2·10^−6^·°C^−1^, respectively. These values are on average slightly lower than the results reported in the literature, with LTEC values for porous mullite ceramics ranging from 5.0 to 5.9·10^−6^·°C^−1^ (between 30 °C and 1000 °C) [2] and are similar to bulk 100 pbw mullite ceramics [65]. The softening of undoped samples begins after 950 °C, but in the case of doped samples, softening begins after 1150 °C.

Therefore, the use of TiO_2_ decreases the LTEC of the investigated ceramic. Softening for both samples occurs due to the presence of amorphous phases, which melt when exposed to a high temperature. According to the LTEC curves, it can be seen that a glassy phase is formed in both samples, which indicates that sintering is taking place with the presence of a liquid phase. Since the amorphous phase consists mainly of SiO_2_, it can also be concluded that the system SiO_2_-TiO_2_ (amorphous phase) is more viscous, with a higher melting temperature than the pure SiO_2_ amorphous phase. Therefore, the glassy phase in samples with 1 pbw of nanosize TiO_2_ has a higher viscosity (as evidenced by the flat softening excess compared to the rather sharp one without TiO_2_). Therefore, it can be assumed that TiO_2_ is most likely in the amorphous phase.

## 4. Conclusions

The influence of the microsize and nanosize TiO_2_ on the mullite formation and on the properties of the porous mullite-alumina ceramic were investigated. The slip casting of the raw material suspension and the use of Al paste as a pore-forming agent for porous ceramic manufacturing is effective and easy to implement, requires low-energy input and material consumption, and is environmentally friendly as no CO_2_-emitting substrates are used. The high-porosity modified mullite-alumina ceramics, with the apparent porosity of 52–69 ± 1%, water uptake of 33–40 ± 1%, pore size distributions of 0.05–0.8 μm, 0.8–10 μm and 10–1000 μm, and bulk density from 1.15 ± 0.05 to 1.4 ± 0.05 g/cm^3^ were obtained due to modification with TiO_2_, compared to others manufacturing methods. The mullitisation intensifies with the increased quantity of used microsize TiO_2._ The use of 2.5 pbw and 5 pbw of microsize TiO_2_ additive and sintering at 1500 °C with a holding time of 1 h, significantly and proportionally increases the content of the mullite phase (60% and 85%) in obtained ceramic samples. XRD analysis demonstrates the increase in the mullite phase peak intensity and the decrease in corundum intensity with the increase in the microsize TiO_2_ amount. The microstructure of the synthesized samples is granular. Elongated mullite crystals are formed with the increased use of microsize TiO_2_, as well as after the use of a small amount (only 1 pbw) of nanosize TiO_2_. The use of the low quantity (1 pbw) of nanosize TiO_2_ additive for mullite ceramic modification improves the mullitisation, as confirmed by XRD analysis, increasing the porosity of the samples (about 69%), and decreases the bulk density to ≈1.3 g/cm^3^ and LTEC to 5.2·10^−6^·°C^−1^ (between 950 °C to 1150 °C). The high porosity and characteristic elongated pore structure can facilitate the use of such porous ceramics as filtration membranes, catalyst carriers and thermal insulators.

## Figures and Tables

**Table 1:** Raw material compositions.

	Samples	Raw Materials, Pbw							Sintering Temperature, °C
		Kaolin	α-Al_2_O_3_	γ-Al_2_O_3_	SiO_2_	Al paste	TiO_2_, μm	TiO_2_, nm	
1.	AK1.4	30	14.65	43.95	11.4	0.2	-	-	1400
2.	AK1.5						-	-	1500
3.	AK2						1	-	1500
4.	AK3						2.5	-	
5.	AK4						5	-	
6.	AK5						-	1	

**Table 2:** Phase compositions of the samples.

Composition	Mullite (%)	Corundum (%)	Cristobalite (%)
AK1.4	26	70	4
AK1.5	42	58	-
AK2	35	62	3
AK3	60	40	-
AK4	85	15	-
AK5	44	56	-

**Table 3:** Comparison of properties of porous ceramics prepared using different pore-forming agents.

Method/ Pore-Forming Agent	Materials	Sintering Temp. (°C)/Holding Time (h)	Porosity (%)	Average Pore Size (μm)	Shrinkage (%)	Bulk Density (g/cm^3^)	Pore Structure
Sacrificial template method/NH_4_HCO_3_ as a sacrificial material [58]	MgO, TiO_2_, Cr_2_O_3_, ZrCl_2_O·8H_2_O, Al_2_O_3_, and SiO_2_	1600	53.9%	20–30	21–22	2.8–3	Rounded pore distributions exhibit remarkable uniformity
Gelcasting/freeze-drying [59]	Polycrystalline mullite fibres, ρ-Al_2_O_3_, alkaline SiO_2_ sol	1400/4	55.95 –64.69	11–43	1.51 –2.03	1.109–1.385	Pores as narrow slit channels
Digital light processing (DLP)/polymerization reaction—form network chains. [60]	Porous mullite ceramic, ZrSiO_4_ additive	1600	74.51	-	21	0.80	Porous membrane
Gelcasting/burning additive [61]	Mullite micro-powder, lignin fibres	600/1 and 1500/2	45.07	9.58	8.85	1.48	Irregular shaped interconnected pores

## Data Availability

The original contributions presented in this study are included in the article. Further inquiries can be directed to the corresponding author.
